# (How) Does Affect Influence the Formation of Habits in Exercise?

**DOI:** 10.3389/fpsyg.2020.578108

**Published:** 2020-10-23

**Authors:** Susanne Weyland, Emily Finne, Janina Krell-Roesch, Darko Jekauc

**Affiliations:** ^1^Department of Health Education and Sport Psychology, Institute of Sports and Sports Science, Karlsruhe Institute of Technology, Karlsruhe, Germany; ^2^Department Prevention and Health Promotion, School of Public Health, Bielefeld University, Bielefeld, Germany

**Keywords:** physical activity, exercise, behavior change, behavior maintenance, habit formation, automaticity, affect

## Abstract

**Objectives:** Habitually instigated exercise is thought to increase health behavior maintenance. Previous research has explored several aspects of habit formation. However, there is a lack of longitudinal research investigating affective determinants, especially post-exercise affective states. Therefore, the present study aimed to investigate (a) if behavior frequency will enhance automaticity, (b) if positive affect will enhance automaticity, and (c) if positive affect will moderate the relationship between behavior frequency and automaticity.

**Methods:** 226 participants (64% females, mean age 24 years) who attended weekly sports and gym classes at two universities were followed for 13 weeks. Class attendance was documented on a weekly basis (behavior frequency) during the semester. Before, during and immediately after each class, participants filled in the Feeling Scale (affective valence). Furthermore, at the beginning of each class, they answered a question about their automaticity in arriving at the decision to attend the class (instigation habit). We used a two-level modeling approach to predict subsequent automaticity by the different constructs at the previous attendance.

**Results:** The cumulative frequency of prior class attendance did not significantly enhance the automaticity of the decision to re-attend the class. There were significant effects of valence on automaticity on the between-subject level, i.e., a one-point higher mean valence score was associated with a 0.62 point increase in automaticity (*p* = 0.001). No moderation effects of affect on the association between behavior frequency and automaticity were observed.

**Conclusion:** Behavior repetition, albeit not significant, and positive affective states at the end of an exercise class may be beneficial in building exercise instigation habits. Practitioners and researchers alike may thus want to emphasize the importance of behavior repetition and affective response for health behavior maintenance.

## Introduction

“The first letter of the psychological alphabet is A for Attitude.”—This statement by Jung, quoted by Hamilton (1929, p. 126), puts the cognitivist paradigm, which later dominated psychological research, in a nutshell. However, since authors like Ekkekakis and Zenko (2016) propose the “escape from cognitivism,” one might consider that A stands for Affect. In the context of physical activity (PA), affect plays a key role and increasingly gains attention among researchers and practitioners alike. On the one hand, affect serves as a motivator of behavior (Finucane et al., 2003) and is involved in the process of PA behavior maintenance (Rhodes and Kates, 2015), and on the other hand, PA can influence affect in both negative (Ekkekakis et al., 2008) and positive (Ekkekakis et al., 2000; Hogan et al., 2013) ways. This study focuses on the role of affective states in the formation of habitual instigation of exercise. Affective states subsume the whole range of states based on core affect (Scherer, 1984; Ekkekakis, 2003), which is defined as “the most elementary consciously accessible affective feelings (and their neurophysiological counterparts) that need not be directed at anything” (Russell and Barrett, 1999, p. 806). Thus, these rapidly and automatically occurring feeling states (Slovic et al., 2007), with the two dimensions valence (pleasure/displeasure) and arousal (low/high) (Russell, 1980), differ from emotions (Ekkekakis, 2003). The broader and general term “affect” refers to any other valenced responses in the global domain of affective feelings (Ortony et al., 1987).

In addition to a potential positive impact on affect, several other benefits of PA with regard to psychological variables have been reported. For example, there is evidence that regular PA reduces levels of stress and anxiety as well as incidence rates of depression, and improves overall psychological well-being (Goodwin, 2003; Ströhle, 2009; Rebar et al., 2015; Rhodes et al., 2017). Furthermore, current research demonstrates that regular PA is associated with the prevention of over 25 chronic medical conditions (Warburton et al., 2007; Garber et al., 2011). Nevertheless, about 31% of adults worldwide are physically inactive (Hallal et al., 2012) and only an alarming 22.6% of adults in Germany meet the WHO recommendations for aerobic and muscle strengthening PA (Finger et al., 2017), even though many individuals may have the intention to be physically active. For example, a recent study showed that 90% of participants intended to engage in moderate PA for at least 150 min per week (de Bruijn et al., 2009). This failure to translate intentions into behavior is a phenomenon referred to as intention-behavior-gap, which reflects “the black-box nature of the underlying psychological process that leads from intention to action” (Sniehotta et al., 2005, pp. 143–144). In their meta-analysis, Rhodes and de Bruijn (2013) quantified the intention-behavior-gap by showing that only 42% of “intenders” acted on their PA intentions. Also, interventions that focus on enhancing intentions thereby promoting behavior change have limited success (Webb and Sheeran, 2006; Rhodes and Dickau, 2012). Thus, there is not only an urgent need to make more people cognitively aware of the health benefits of sustained PA, but to also help them to successfully carry out an intended behavior, such as engaging in PA. Focusing on intention as the proximal determinant of behavior, as postulated in traditional social-cognitive models like the Theory of Planned Behavior (TPB; Ajzen, 1985), may not be sufficient in explaining actual behavioral instigation and regular execution. Rather, automatic processes need to be additionally considered (de Bruijn and Rhodes, 2011). The two pathways are summarized in dual process theories such as the Affective-Reflective Theory (ART) of physical inactivity and exercise. The ART was developed by Brand and Ekkekakis (2018) to explain the initiation of exercise-related actions or the persistence of physical inactivity. According to the theory, a fast type-1 process leads to an action impulse via automatic associations and automatic affective valuations (Antoniewicz and Brand, 2016), and a slower type-2 process can result in action plans via reflective evaluation provided that self-control resources are available (Baumeister and Heatherton, 1996). Explaining their PAAM model that identifies predictors of PA adoption and maintenance, Strobach et al. (2020) argue that the control of behavior gradually shifts from being explicitly to being implicitly controlled when it is repeated under stable contexts due to habit formation. One study found that past exercise behavior had a significant positive effect on the intention to continue exercising during the next 6 months, thus stabilizing it, while at the same time past behavior did not exhibit a significant indirect effect via intention on future behavior, but had a strong direct effect (Rodrigues et al., 2019). In sum, one of the implicit constructs that should be considered with regard to the intention-behavior-gap is habit (de Bruijn and Rhodes, 2011).

Gardner and Lally (2018, p. 207) define habit as “a process whereby encountering a cue triggers an impulse to perform an action that has, through learning, become a learned response to the cue.” In order to develop a method of measuring habit, Verplanken and Orbell (2003) summarize the basis features of habit as follows: previous repetition of the behavior; and features of automaticity, namely difficulty of overruling strong habits, lack of awareness, efficiency; and their reflection of someone’s identity. Thus, automaticity is a main characteristic of habit (Aarts and Dijksterhuis, 2000; Hagger, 2019). Assuming that habit automaticity is cue-dependent (Orbell and Verplanken, 2010; Wood and Rünger, 2016), once behavior has become habitual it is supposed to be insensitive to lack of motivation (Rebar et al., 2019; Gardner et al., 2020). In their recent meta-analysis, Gardner et al. (2011) found a medium-to-large correlation between habit and behavior, suggesting that habit explains for about 20% of variance in those health-related behaviors. Combining these two effects of habit on behavior, namely bridging dips in motivation and a correlation between habit strength and behavior frequency (Gardner et al., 2012; Rebar et al., 2016), it is possible that establishing habits might facilitate behavior maintenance. The underlying assumption is that the habit process may trigger selecting an action out of several behavioral alternatives. This habitual selection of an action for performance is defined as habitual instigation (in contrast to habitual execution, which means habitually performing the already chosen behavior) (Gardner et al., 2020). In a randomized controlled trial examining the effect of a workshop on establishing a preparatory exercise habit, the experimental group indeed showed a significant increase in physical activity, use of cues and practice consistency compared to the control group (Kaushal et al., 2017).

Theoretically, habits are easily developed, as repetition of behaviors in stable contexts might be sufficient to strengthen links between salient cues and subsequent actions in associative memory, which may in turn result in highly accessible context-behavior associations that speed up enactment (Verplanken, 2006; Danner et al., 2008; Gardner and Lally, 2018; Hagger, 2019). However, reality is more complex. In their attempt to model habit formation in the real world, Lally et al. (2010) asked volunteers to repeat a self-chosen health behavior in the presence of a cue of their choice and to report automaticity on a daily basis. An asymptotic curve reflected the process of habit formation, assuming that automaticity increases rapidly with every repetition in the first days while additional gains then decelerate over time. Finally, habit formation reaches a point where growth in automaticity is no longer possible despite maintaining repetitions. This model was valid for 62 of the 82 participants, which indicates that repetition of behavior was sufficient to form automaticity in these 62 individuals. However, there was variation in the absolute level of achieved automaticity and the number of days needed to reach this individual maximum. Thus, rates of automaticity formation are highly variable albeit an equal number of repetitions, leading the authors to conclude that the final habit strength is not exclusively determined by repetition. While it is possible that anticipated affect or intrinsic rewards played a role in the participant’s choice of the health behavior, the study reveals no information about affect itself. In the present study, the research question was whether affective states is another variable that influences habit formation.

Conceptually, determinants of habit formation can be categorized into variables influencing the intention to act, the likelihood of acting on intentions, the motivation to maintain a behavior after successfully initiating it, or the development of cue-action associations (Gardner and Lally, 2018). As for affect, this intrinsic rewarding outcome is supposed to play a role on multiple levels: First, it may lead to more frequently performed behavior and sustained motivation which in turn may prompt maintenance and habit development. This assumption is based on a psychological hedonism of the past which is associated with learning theories (Insko and Schopler, 1972). An example is the “law of effect,” which was developed by Thorndike (1911). His animal-learning studies led him to conclude that a behavioral response to a cue will be more likely to be shown after encountering the stimulus again in the future, if the behavior was followed by satisfaction. Hedonism of the past, in general, states that individuals engage in behavior that maximized reward and minimized displeasure in the past (Insko and Schopler, 1972). In fact, individuals having a more positive affective response during acute moderate-intensity exercise were more active in the future (Schneider et al., 2009). Second, affect may increase or expedite context-behavior associations. This assumption is based on a premise resulting from a combination of hedonism of the past and a stimulus-response approach: When the affective response to a cue-response situation is pleasurable, a learned association between stimulus and response will be formed (Insko and Schopler, 1972). In the law of effect, a positive correlation between satisfaction and the resulting strengthening of the bond is assumed (Thorndike, 1911). In line with this, the Associative Cybernetic Model proposes that once an outcome is rewarding, the signal to habit memory, which strengthens the stimulus-response relationship, will be supported (de Wit and Dickinson, 2009). Consequently, this process reinforces the contribution of each rewarded behavior performance to habit formation (Wiedemann et al., 2014) and can therefore explain different curves of habit formation despite comparable behavioral frequency. Thus, affect—especially during exercise—is supposed to influence habit development not only due to repetition of the behavior but also via the reinforcement of the relationship between behavioral repetition and habit strength.

**FIGURE 1:**
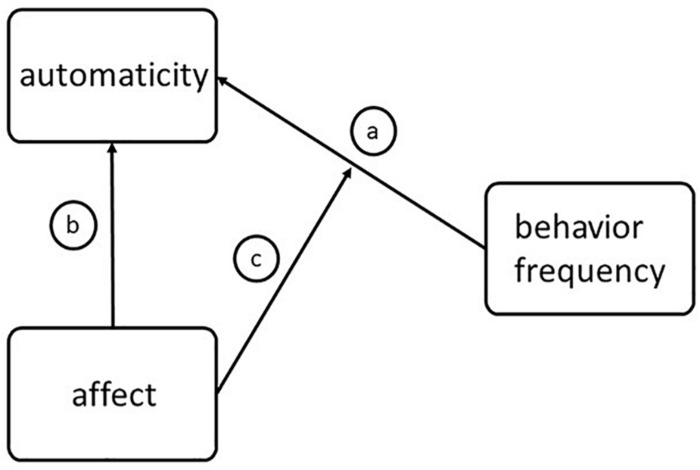
Hypotheses.

Investigating determinants of habit strength, one cross-sectional study found an interaction between motivational regulation and past behavior (Gardner and Lally, 2013). The authors hypothesized that past behavior may be a stronger predictor of habit strength among intrinsically motivated participants, suggesting that enjoyment derived from autonomously motivated PA may strengthen the relationship between past behavior and habit development even more. In line with this, another study investigated intrinsic rewards such as enjoyment and found that intrinsic rewards predict exercise frequency via habit strengths for maintainers (and via behavioral intentions for initiators) (Phillips et al., 2016). Furthermore, Kaushal and Rhodes (2015) investigated the influence of affective judgments about exercise on habit formation in a longitudinal study among new gym members, and reported that affective judgments at baseline were the main predictor of habit development. The authors concluded that a reward like positive affect increased the likelihood of an individual performing the behavior again without conscious deliberation. However, the study had several limitations, i.e., the first follow-up assessment of habit scores was done after 6 weeks; and, in particular, affective judgments refer to beliefs or expectations about affect and are therefore not affective responses *per se* (Ekkekakis et al., 2018). Overall, only few long-term studies that examined affective determinants of habit formation are available, especially in the context of physical exercise. Therefore, the purpose of the present longitudinal study was to examine the role of affective states and behavior repetition in the formation of real-world exercise instigation habits among adults. Since it is recommended in the literature to not only analyze affective changes in group means, but also at an individual level, we explored affective states on both between-person and within-person level (Ekkekakis, 2008). We hypothesized that (a) behavior frequency will enhance automaticity, (b) positive affect will enhance automaticity, and (c) positive affect will moderate the relationship between behavior frequency and automaticity (see Figure 1).

## Materials and Methods

### Participants

Participants were university students or employees who participated in 10 sports and gym classes at no or low cost during the winter term 2015/16. These courses are unconditionally offered each term to all students or employees of the universities. As the limited available spots were assigned by applying the “first come first served” principle, interested persons had to register for specific courses ahead of time. Each specific class started at the beginning of the lecture period and there was a great variability in date, coach and participants from semester to semester. Given this fluctuation, the participants may have attended a similar course, but they cannot have attended the identical course on the same date, with the same instructor, the same co-participants nor at the same sports facilities as before. Therefore, context-specific cues that may have been associated with the exercise behavior before change. We consequently assume that participants started the class with no habit to attend this specific class and are appropriate for studying the development of a new habit. We included classes with a medium size (about 15–30 participants) and an adequate practice time (about 60–90 min). Thus, our study sample can be regarded as a convenience sample, although participants were not self-selected as throughout the courses nearly all consented to participate in our study. This approach led to a total sample of 145 female und 81 male (*N* = 226) university students and employees, who provided sufficient complete data and were included in the presented analyses. Course instructors of the 10 classes were informed prior to the study about the design and aims, and all instructors gave their consent to participate in this research. Study participation for participants of the sports classes was voluntary and interested individuals were asked to provide written informed consent, which was done by nearly all of potential participants. However, we have no information on the number of individuals who refused to participate, as the complete list of participants attending the sports classes was not available to our research team due to data protection policies. The study was approved by the Data Security Commissioner and the Ethics Committee of one of the universities.

### Design and Setting

In order to study the influence of affective states and behavior frequency on automaticity formation on a between- and within-person level, this study had a longitudinal design with weekly measurement time points. It was conducted at two German universities during the winter semester 2015/2016 (October 2015 to February 2016). Course duration varied slightly depending on the length of the semester at each university (ranged from 13 to 15 sessions; for comparability, only the first 13 weeks were included in the analyses), and no classes took place during the 2-weeks Christmas holiday break. The number of weeks needed to form a habit is highly variable (for an overview see Hagger, 2019), but since evidence suggests that attendance in the first 5 weeks is crucial for habit formation (Armitage, 2005), we consider the time span of one term to be sufficient. The study settings were sports and gym classes during which participants carried out various types of aerobic exercise, including dance-related exercise (Zumba, Bokwa), martial arts (Kickboxing, Taekwondo, Capoeira), Freeletics (a specific set of endurance and strength exercises), and basketball training.

### Procedure

Individuals who agreed to participate in the study signed a consent form during their first attendance of the course. Participants then completed a baseline questionnaire to report past exercise behaviors and habit strength (please refer to section “Baseline Questionnaire”). Student assistants attended all selected courses on a weekly basis. At the beginning of a course, they documented participation and handed out a short questionnaire measuring affective states and automaticity to all attending study participants. After approximately half of the class time (after about 45 min), and immediately after the training, the same short questionnaire was again provided to participants. After each class, the student assistants regathered all questionnaires. In order to collect the data pseudonymously, each participant had an individual code, consisting of letters and numbers derived from family names, year and place of birth. This enabled the lead investigator to match the questionnaires to each participant.

### Measures

Participants filled in a baseline-questionnaire during their first week of attendance and, every week they attended the class, a short weekly questionnaire at three time points: at the beginning of the training, approximately after half of the class time, and immediately afterward. In the following, only measures relevant for the present analyses are described.

#### Baseline Questionnaire

##### Sociodemographic Information

Sex (male, female), age (in years), and student status (student yes/no) were collected.

##### Past Exercise Behavior

To adjust for past behavior, participants were asked whether they had already been exercising on a regular basis (yes/no) before registering for the class. If they responded with “yes,” they were asked to provide information on how long they had been exercising on a regular basis (in months or years). Exercise was defined as any leisure time activities that included physical exercise regardless of whether these activities were performed alone, in a team, or a sports club, and examples were given (e.g., team sports either within or outside of a club, walking, swimming, horse riding, etc.). Mainly sedentary sports like chess, computer games or fishing were explicitly excluded from the definition.

##### Habit Strength

To measure general exercise instigation habit strength, the Self-Report Habit Index (SRHI; Verplanken and Orbell, 2003) was completed by study participants. However, three items on frequency of behavior from the original 12-item measure were excluded, as they have been subject to discussion in literature (Gardner, 2015) and since leaving them out did not change the main results of the original scale development studies (Verplanken and Orbell, 2003). For the remaining nine SRHI-items, the wording of each item stem was “To go exercising is something…” and ended, for example, in one item at “… is something I do without thinking.” Therefore, the scale rather taps the decision to go exercising than the execution of a specific exercise behavior (for a distinction between instigation and execution habit see the response to Hagger by Gardner et al., 2020). The scale showed a very good internal consistency of alpha = 0.917 and was approximately normally distributed. The scale did not address the same decision/behavior as the short weekly questionnaire. As habit strength for instigation habit was measured before the weekly course started, measuring the habit to attend exactly this course would not have made sense.

#### Weekly Short Questionnaire

##### Attendance

The weekly attendance of each participant was recorded by a student assistant who attended every session (1 = present, 0 = absent, or missing when class did not take place). As a measure for frequency of behavior, we built a variable that indicated number of prior class attendance for every week. That is, for someone who attended the class for the second time, the variable “frequency of attendance” had a value of 1. We also coded the length of the interval until an individual attended the class again, with the unit of measurement being the opportunities to participate (since there were instances where a class did not take place for 1 week). For someone who came back regularly the next time, the length of time was coded 1, for someone who missed one opportunity before they came back, the length was coded as 2, and so on.

##### Affective State

Current affective states were measured by two items based on Russell’s affect circumplex model (Russell, 1980). According to the model, two dimensions of affect need to be distinguished, namely affective valence and energetic arousal. Affective valence was measured through the Feeling Scale (Hardy and Rejeski, 1989). The question “How do you feel at this moment?” was answered on a scale of 1 (very bad) to 10 (very good). In the original version, response options range from −5 to 5, but we modified it to range from 1 to 10 to better align with other scales used in this research. Energetic arousal was measured by the Felt Arousal Scale (Svebak and Murgatroyd, 1985). The item read “How aroused do you feel at this moment?” and was answered on a 10-point scale of 1 (extremely tired) to 10 (extremely energized). According to Backhouse et al. (2007), the two scales have been widely applied and showed both satisfactory convergent and discriminant validity. Additionally, as further predictor of positive affect the increase in affective valence from the start to the end of the class (valence end minus valence start) was used. Positive values reflect an increase in valence during the class.

##### Automaticity

On a weekly basis, automaticity was measured at the beginning of the class. Participants were asked to rate how strongly they agreed with the following statement on a scale of 1 (not at all) to 10 (absolutely): “I arrived at the decision to attend the class today completely automatically (without thinking).” This single automaticity item is based on a similar measure employed by White et al. (2017) and derived from the automaticity subscale of the Self-Report Habit Index (SRHI) (Verplanken and Orbell, 2003; Gardner et al., 2012). This single item has shown adequate content and predictive validity (Verplanken and Orbell, 2003; Gardner et al., 2012), and was therefore chosen in order to keep the weekly questionnaire short for reasons of feasibility. The phrasing of the item is consistent with the concept of instigation (in contrast to execution) habits (Gardner, 2015). The decision to exercise is an important element of exercising behavior (Verplanken and Melkevik, 2008).

### Statistical Analysis

Data are described as means (M) and standard deviations (SD) for continuous variables and number (N) and percentages (%) for categorical variables.

Automaticity at a given participation week was predicted by affective state at the preceding participation in the exercise class. As the weekly data was nested within individuals, we used a two-level modeling approach, employing Mplus version 8 (Muthén and Muthén, (1998-2015)).

At the within-person level, automaticity was predicted by preceding affective valence, changes in affective valence during the preceding class, the cumulative frequency of subsequent participation, the length of the period from last attendance at the exercise class as well as the interaction between affect and frequency. Since automaticity was measured at each participation, the preceding automaticity was also included as predictor to adjust for autocorrelation over time.

**FIGURE 2:**
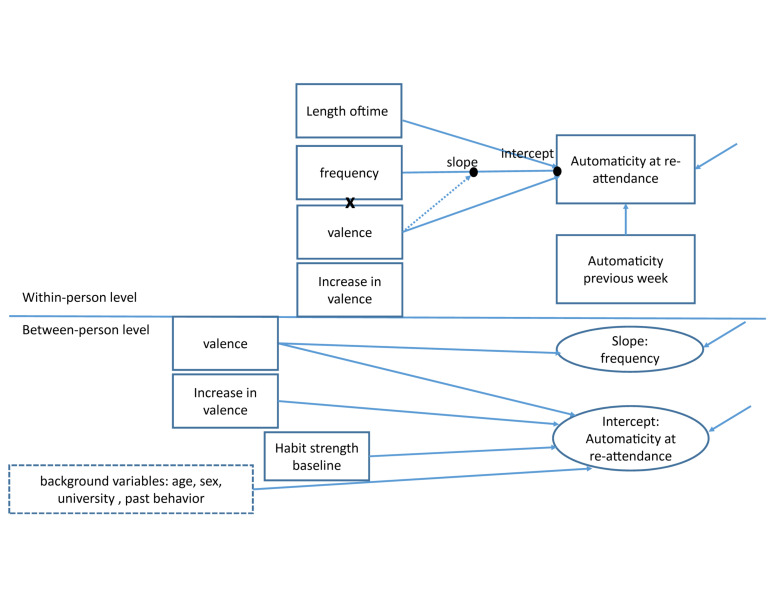
Final model with interactions.

As a predictor at the between-person level besides affect, baseline habit strength was used to adjust for differences in habit base level. Different sociodemographic measures were tested as predictors and included when meaningful. See Figure 2 for the final model with interactions.

Research shows that positive changes during exercise are relevant for future exercise behavior (Schneider et al., 2009; Rhodes and Kates, 2015), however, contextual limitations lead us to conclude that we were not able to detect those dynamic changes (please refer to section “Discussion”). Due to high correlations of the three items which were completed at different times during the class, we could not include all of them in our model (see Tables 1–3 for correlations). Rather, we used the measurement at the end of the class as well as the change in affect from beginning to the end as manifest variables in the prediction. Arousal and valence are seen as two orthogonal dimensions in the circumplex model. However, we found that both were highly correlated (*r* = 0.714) and could not be used in the same model because of multicollinearity. We therefore decided to examine both affect dimensions in separate models. However, in line with Feldman Barrett (Barrett, 2006), we suppose that valence is the most basic building block of emotional life and, therefore, expect it to have a greater influence on habit formation via motivational processes than energetic arousal, which may rather indicate the intensity of valence. Thus, the results presented here only refer to valence.

**TABLE 1:** Correlations: within-person level.

	SA	PA	AV3	CF	DR	DV
SA	1					
PA	0.292	1				
AV3	0.032	0.073	1			
CF	0.101	0.140	−0.037	1		
DR	–0.074	–0.112	−0.029	−0.080	1	
DV	–0.019	–0.096	0.626	0.033	0.041	1

*SA, subsequent automaticity; PA, preceding automaticity; AV3, affective valence (end of class); CF, cumulative frequency of participation; DR, duration until re-attending; DV, difference valence (increase in valence during participation in exercise class). The sample statistics for within and between refer to the maximum-likelihood estimated within and between covariance matrices, respectively.*

**TABLE 2:** Correlations: between-person level.

	HB	AGE	SEX	PB	UNI	SA	AV3	DV
HB	1							
AGE	0.158	1						
SEX	–0.151	–0.188	1					
PB	0.435	0.304	–0.149	1				
UNI	0.100	0.220	–0.110	0.253	1			
SA	0.335	–0.101	0.049	–0.030	–0.483	1		
AV3	0.172	–0.094	–0.032	–0.064	–0.329	0.427	1	
DV	–0.029	–0.088	0.112	–0.077	–0.267	0.053	0.400	1

*HB, habit strength baseline (SRHI); PB, past behavior (regular exercising in months); UNI, University (1 vs. 2); SA, subsequent automaticity; AV3, affective valence (end of class); DV, difference valence (increase in valence during participation in exercise class). The sample statistics for within and between refer to the maximum-likelihood estimated within and between covariance matrices, respectively.*

**TABLE 3:** Correlations: descriptive statistics of overall sample.

	HB	AGE	PB	SA	PA	AV1	AV2	AV3	AR1	AR2	AR3	CF	DR	DV
HB	1													
AGE	0.151	1												
PB	0.447	0.315	1											
SA	0.203	–0.053	–0.017	1										
PA	0.167	–0.043	–0.042	0.550	1									
AV1	0.081	0.020	0.030	0.210	0.251	1								
AV2	0.139	0.029	0.005	0.185	0.227	0.510	1							
AV3	0.054	–0.033	–0.019	0.185	0.196	0.370	0.625	1						
AR1	0.103	0.046	0.043	0.239	0.266	0.712	0.399	0.270	1					
AR2	0.131	0.016	–0.003	0.185	0.212	0.454	0.727	0.559	0.447	1				
AR3	0.061	0.002	–0.022	0.163	0.218	0.269	0.509	0.715	0.251	0.630	1			
CF	0.069	0.096	0.027	0.073	0.141	–0.100	–0.093	–0.080	0.034	–0.101	–0.017	1		
DR	–0.055	–0.046	–0.030	–0.090	–0.113	–0.068	–0.017	–0.035	–0.054	–0.029	–0.020	-0.080	1	
DV	–0.026	–0.047	–0.044	–0.029	–0.056	–0.582	0.085	0.540	–0.409	0.078	0.382	0.020	0.031	1

*HB, habit strength baseline (SRHI); PB, past behavior (regular exercising in months); SA, subsequent automaticity; PA, preceding automaticity; AV1-3, affective valence (three measurement time points); AR1-3, arousal (three measurement time points); CF, cumulative frequency of participation; DR, duration until re-attending; DV, difference valence (increase in valence during participation in exercise class).*

Valence was used as a predictor at the within- as well as the between-person level. To this end the variable is decomposed into two latent components: At the between-person level a between covariance matrix is used where the variation between persons is captured by subtracting the overall mean from the latent person mean (grand mean centering). At the within-person level, a pooled within covariance matrix is used where the indicators are implicitly group mean centered. That is, the between component of a person is subtracted from the value at a given occasion.

We excluded those observations where no information on automaticity at the beginning of the class and no information on valence at the end of the preceding class were available. Other missing values were estimated implicitly using the full information maximum likelihood approach implemented in MPlus. Furthermore, we only included data from participants if one participation week was followed by re-attending the class, so that automaticity at the following participation could be predicted.

## Results

### Descriptive Statistics

After the exclusion of missing observations, the final sample consisted of 1,082 observations from 226 individuals. 64.2% of the sample were females, 87.9% were students (percentage of valid answers, 44 were missing), 46% were from university 1, 54% from university 2, and the mean age was 24.46 (*SD* = 5.25) years.

On average, each individual participated 6.8 times (range, 2–13). The intra-class correlation for automaticity was 0.360, that is about 36% of variation was between persons. See Table 4 for descriptive statistics.

**TABLE 4:** Descriptive statistics.

Variable	N	Mean (*SD*)	Min–Max	Median
**Measured between-persons (time invariant)**				
Habit strength baseline	209	4.092 (1.401)	1.00–7.00	4.11
Past exercising (in months)	203	87.394 (94.914)	0–384	36
**Measured within-person (time-varying, N is overall number of observations)**				
Automaticity (at subsequent participation)	1,082	6.893 (2.941)	1.00–10.00	8.00
Automaticity (at preceding participation)	1,076	6.573 (3.127)	1.00–10.00	8.00
Frequency of attendance before (accumulated number of attended classes)	1,082	2.987 (2.640)	1.00–12.00	2.00
Duration until re-attendance (opportunities, generally equals weeks)	1,082	1.471 (1.048)	1.00–12.00	2.00
Increase in valence from beginning to end of class	1,045	0.917 (1.850)	−6.00–9.00	1.00
Valence (at end of class session)	1,082	7.679 (1.629)	1.00–10.00	8.00
Arousal (at end of class)	1,055	7.317 (1.862)	1.00–10.00	8.00

*Overall, 1,082 observations from 226 persons were included. Number of observations for individual variables differs due to missing values.*

### Prediction of Automaticity

The candidate models for the prediction of automaticity at subsequent participation were tested in a stepwise manner. We first analyzed if age, sex, past behavior, and university predicted automaticity and, therefore, should be included as confounders. The only variable with a significant effect was university: the automaticity value from the university 2 subsample was estimated 1.520 points lower on average than at university 1 (*p* < 0.0001). All other potential confounders were not significant and thus not included in the final models. The variable university, however, distorted the model. We tested interaction effects with the other predictors of relevance. None of these interactions were approaching significance. We therefore decided to also exclude university as a predictor in favor of a more precise estimation. A model with age, sex, past behavior, and university had larger BIC (31632.3) and AIC (31402.9) values than the model without these background variables. Hence, we proceeded with the more parsimonious models without the tested background variables.

As described before, we restricted our models on valence as affective state variable, since including arousal caused multicollinearity and large standard errors. The separate model for arousal (not shown) achieved essentially the same results as the separate model for valence which is presented here.

The results of SEM models are shown in Table 5. We first tested the model with main effects only and then entered the interactions between number of sessions attended before (frequency) with valence on both the within- and the between-person level.

**TABLE 5:** Results of the prediction model for valence.

	Model with main effects			Model with interactions		
	Coefficient	*SE*	*p*-value	coefficient	*SE*	*p*-value
**Within-person level fixed effects (weekly fluctuations)**						
Automaticity previous attendance	0.229	0.053	< 0.001	0.210	0.069	0.002
Duration until re-attendance	−0.084	0.080	0.294	−0.085	0.081	0.292
Frequency of attendance before	0.038	0.028	0.169	−0.039	0.177	0.825
Valence end of class	0.028	0.083	0.733	−0.003	0.108	0.978
Increase in valence during class	0.018	0.063	0.778	0.022	0.065	0.740
Valence × frequency	/	/	/	0.011	0.023	0.629
**Between-person level fixed effects**						
Habit strength baseline	0.298	0.090	0.001	0.303	0.091	0.001
Valence end of class	0.623	0.195	0.001	0.639	0.229	0.005
Increase in valence during class	−0.367	0.181	0.042	−0.376	0.189	0.047
Cross-level interaction: Valence x frequency (slope)	**/**	**/**	**/**	−0.009	0.039	0.819
**Random effects (variances)**						
Residual variance automaticity within	4.438	0.408	< 0.001	4.316	0.414	< 0.001
Residual variance automaticity between	1.747	0.454	< 0.001	2.019	0.773	0.009
Slope frequency	/	/	/	0.014	0.034	0.671
**Model fit information**						
LL	−13499.424			−13499.027		
AIC	27058.849			27066.053		
BIC	27208.446			27235.597		

*Results from Mplus 8 using full information maximum likelihood estimation for cases with missing values. N = 1,082 observations from 226 persons, results of maximum likelihood estimation with robust standard errors. SE, standard error; LL, Log-likelihood; AIC, Akaike’s information criterion; BIC, Bayes information criterion.*

As can be seen in the right columns of Table 5, the interactions between cumulative frequency of participation with valence were not significant, neither at the within-person nor the between-person level. Both models did not differ in terms of a chi-square difference test [χ^2^_(df = 4)_ = 0.69, n.s.], but AIC and BIC values preferred the model with only the main effects. Although the estimated coefficients of both models were very similar, we focus on the results of the main effect model.

On the within-person level, automaticity was only predicted by preceding automaticity. None of the other predictors were significant. On the between-person level, we found automaticity to be predicted by baseline habit strength, preceding valence, and the change in valence during the preceding class.

#### Prediction of Automaticity by Behavior Frequency

Cumulative frequency of prior class attendance as a measure of behavior repetition was not associated with an enhanced automaticity of the decision to re-attend the class (as indicator of habit strength).

There was a significant correlation between frequency and preceding automaticity (regression coefficient for frequency = 0.157, *p* < 0.001). In a model without preceding automaticity, frequency was significantly associated with subsequent automaticity (coefficient = 0.077, *p* = 0.026) although the effect was also small (overall model results not shown).

#### Prediction of Automaticity by Affective States (Valence)

There was no association between valence and enhanced subsequent automaticity at the within-person level. This indicates that there was no change in automaticity for an individual after weeks where valence was especially high compared to other weeks. The same was true for an increase in valence during the preceding class, which also showed a non-significant effect on subsequent automaticity. However, significant associations were found at the between-person level. Individuals with a higher average valence (higher mean values over the weeks when participating in class) had higher automaticity values than those with lower mean valence, with a one-point higher mean valence score associated with a 0.62 point increase in automaticity (both measured on the same scale, *p* = 0.001). This effect was present after accounting for baseline habit strength as well as preceding automaticity. In terms of changes in valence during the class, our result pointed to persons with a higher average increase in valence during class, showing smaller automaticity values when re-attending (per one-point-increase expected automaticity went down by 0.37, *p* < 0.05).

#### Prediction of Automaticity Through the Interaction of Frequency and Valence

There was no moderating effect of affect on the relationship between behavioral repetition and automaticity, as indicated by non-significant effects in the model with interactions on either level (Table 5). Neither was the association between behavioral frequency with automaticity strengthened after weeks when valence was higher, nor was this expectation approved between persons, that is, persons with higher average valence over the term did not show larger associations between behavioral frequency and subsequent automaticity.

## Discussion

The purpose of this study was to examine the role of affective states and behavior frequency in the formation of real-world exercise instigation habits among adults. Overall, it could be shown that positive affect was significantly associated with subsequent automaticity, whereas behavior frequency did not significantly predict subsequent automaticity, and that affect did not significantly moderate the relationship between behavior frequency and automaticity.

With regard to our first hypothesis that behavior repetition will enhance automaticity, we did not observe a significant effect of frequency on automaticity. Two aspects need to be critically mentioned here. First, behavior frequency was rather low as participants attended the class on average only seven times (range 2–13). How long it takes to establish a habit is discussed in the literature (Walter, 2018; Hagger, 2019). In line with this discussion it is possible that in our study, behavior repetition did not occur often enough to enhance automaticity. Second, despite the non-significant prediction of automaticity, there was a significant correlation between frequency and preceding automaticity (about twice as large as with predicted automaticity at the within level), which might point to preceding automaticity masking the effect of frequency. In a model without preceding automaticity, frequency was significantly associated with subsequent automaticity although the effect was also small. Furthermore, in the model presented here, there was a minimal increase in the expected direction, i.e., for each exercise class visited, the resulting automaticity increased slightly. Additionally, we found the duration until re-attendance to be slightly negatively associated with automaticity. These tendencies are in line with other findings that support the role of behavior frequency for the formation of habits. One study that explored habit formation in a real-world setting found that repeating a behavior in a stable context increases automaticity (Lally et al., 2010). An asymptotic model best reflected the process of habit formation for 62 out of 82 individuals, and those study participants for whom this model provided a poor fit had shown lower behavior frequency during the time of the study. The finding that repeating a behavior leads to greater automaticity scores is also in line with the habit theory that suggests that habits are developed through the strengthening of a cue-behavior relationship (Verplanken, 2006; Gardner and Lally, 2018). Therefore, this cue-behavior association needs to be encountered at all which requires the enactment of a behavior when confronted with the cue and, in order to gain a degree of automaticity, needs to be repeated.

The second hypothesis stated that positive affect will enhance automaticity. Significant relationships were found for affective valence on the between-person level, but not on the within-person level. The non-significant effect on the within-level indicates that after weeks in which the valence score of a person at the end of class or the increase of affective valence during class was higher than usual for this person, there was no increase in the resulting automaticity. However, we found two significant effects on the between-person level and thereby added new insights on the role of affective states on automaticity development to the literature. First, for affective valence at the end of the class, the effect on the between-person level suggests that people who on average reported higher values in valence at the end of the class also had higher automaticity scores. One explanation for this is that individuals repeated the behavior more often because of the positive affect they associated with it and therefore built stronger habits. Theoretically, this assumption is supported by psychological hedonism of the past which states that formerly rewarded behavior is repeated more often in the future (Insko and Schopler, 1972). In their review, Ekkekakis and Dafermos (2012) concluded that affective responses to exercise, although measured in various ways due to methodological diversity, in fact predict subsequent exercise behavior. One study measured affective valence during and immediately following a brief treadmill walk at two time points (6 months apart) and found that affect reported during the walk was cross-sectionally and longitudinally associated with physical activity (Williams et al., 2012). Another study found that the relationship between intrinsic exercise rewards (such as enjoyment) and exercise behavior can be explained differently depending on the stage of adoption (Phillips et al., 2016). For initiators, this relationship was mediated by intentions, whereas it was mediated by habit strength for longer term exercisers (maintainers). Given that the participants in this study were unexperienced in terms of the specific instigation behavior, it is possible that positive affect strengthened their intentions to attend the course again. Due to the non-significant effect of frequency on automaticity, however, we cannot confirm that affect influences habit strength via behavior frequency. Therefore, other explanations are also possible, one of them being the possibility of a direct influence of affect on habit formation independent of behavior repetition and another one being methodological artifacts. We measured the two implicit constructs automaticity and affect on a weekly basis, one after the other, in one questionnaire. Depending on the answer a participant gave to the question of automaticity, they may have drawn conclusions about their affect, similar to what Gardner and Lally (2013, p. 494) call “a *post hoc* self-perception process.” So possibly, habitual exercisers inferred positive affect from their habitual behavior whereas non-habitual exercisers reported no or less positive affect. Second, for the increase in affective valence during class, the effect on the between-level suggests that people who had a higher increase in valence had *lower* automaticity scores. Two lines of reasoning lead us to conclude that this result should not be over-interpreted. First, it was impossible to measure affective states multiple times during the exercise class as this would have meant a serious disruption of the flow of participants. We conclude that we were not able to detect dynamic changes in affect during exercise—although being aware that it would be desirable for future research (for recommendations regarding the timing of affect assessment see Ekkekakis et al., 2020). The affect assessment at the end of the class and the difference variable (after minus before) might therefore not reveal the true and differentiated affective response. However, one study also showed the tendency of affective responses *after* a hard-intensity task to be positively associated with future participation (Schneider et al., 2009). It should be investigated whether dynamic changes in the affective response during exercise influence habit formation. Second, further methodological concerns should be mentioned. That is, the affective state at the beginning of the class can be based on various reasons, while the state at the end of the class may more exclusively refer to the sports class itself. Subtracting these values from each other can therefore be problematic, and potential solutions discussed in the literature include a direct comparison operationalization (Peter et al., 1993), e.g., an item that directly asks for affective change.

The third hypothesis stated that positive affect may moderate the relationship between behavior frequency and automaticity. No significant moderation effects could be found, neither on the between-level nor on the within-level, and neither for affective valence at the end of the class nor for the increase of affective valence during class. These findings suggest that the relationship between frequency and automaticity does not differ depending on the degree of valence. In light of the above mentioned non-significant effect of affect on automaticity on the within-level and this non-significant moderation effect, our findings contradict parts of the assumptions of the Associative-Cybernetic Model (de Wit and Dickinson, 2009). The model suggests that there are two ways a reward can have an impact on habits. First, a reward should strengthen habits mediated by behavior repetition. Second, a reward should moderate the relationship between behavior repetition and habit. This could not be shown in our study. We speculate that this is because our study measured affective states *per se*, a mental but not cognitive or reflective phenomenon (Russell, 2003), while other studies that reported a moderation effect operationalized intrinsic rewards as cognitive constructs. For example, one study that confirmed the Associative-Cybernetic Model for fruit and vegetable consumption assessed intrinsic rewards by directly asking the participants whether the consumption was rewarding (Wiedemann et al., 2014). Gardner and Lally (2013) found that prior action was a stronger predictor of habit strength among participants who were of the self-determined motivational regulation type and showed autonomous motivation such as intrinsic interest. In line with our hypothesis on the moderation effect, they speculate that the enjoyment of intrinsically motivated PA may reinforce the past behavior-habit strength relationship. However, they did not measure enjoyment or any implicit constructs.

### Strength, Limitations, and Future Directions

One strength of this study is the weekly measurement of exercise class attendance over a period of 3 months in a relatively large sample. In order to understand habit formation, cross-sectional studies or observations for only a few weeks or at insufficient time points seem to be less appropriate. Further, the longitudinal design allowed us to explore the effects on a between- and within-subject level. Future studies should investigate the relationship between affective states, habit formation, and exercise maintenance by continuous measurement over an even longer period of time than in the present study. This would allow for examining the effects of affective states and habits on long-term adherence. Moreover, exercise class attendance was measured quasi-objectively by weekly observation of attendance so that we can rule out systematic bias of subjective measures of PA (Jekauc et al., 2014). The fact that we measured affective states rather than affective attitudes, affective judgments or anticipated affective responses which are not affective states *per se* (Ekkekakis et al., 2018) is also one of the several merits of this study. If one assumes that affect is not a cognitive or reflective sensation (Russell, 2003), it is not necessary to measure it as a cognitive construct: By asking participants to reflect about their affective attitudes or judgments, however, the answer is the result of cognitive operations (Ekkekakis et al., 2018). Applying the Feeling Scale (Hardy and Rejeski, 1989) and Felt Arousal Scale (Svebak and Murgatroyd, 1985), we are coming closer to measuring affective states *per se* and thereby extend the literature on the role of affect.

This study was an observational one which cannot prove causality, although future events were predicted from preceding ones. A potential shortcoming of the present study is the rather high percentage of missing values. Since we only collected data from those individuals who attended the class, there is no information about the reasons for the absence of the missing participants. Therefore, we do not know whether the missing is random, due to a lack of habit formation or other reasons. One promising approach to gather information about reasons for a dropout are real-time analyses and feedback from wearables (Ebner-Priemer et al., 2019). However, it is possible that the lack of motivation to attend the exercise course is associated with the lack of motivation to participate in the study. Furthermore, the 2-weeks Christmas holidays in the middle of the semester led to a break, which is another limitation. As habits form due to repeated performance in stable contexts (Aarts and Dijksterhuis, 2000; Wood and Neal, 2007), the break might have represented an interruption in habit formation. Again, no data is available of the participants during the break. However, we found no indication of a drop in automaticity after this break. Another, rather controversial limitation lies in the methodology for measuring habits. In this study, baseline habit and weekly automaticity scores were measured by self-report. Whether it interrupts or hinders the formation of habits when weekly questions are asked about the automaticity of a process that is actually supposed to be no longer reflective, can be questioned critically. Also, some scientists have reported that subjective insights into unconscious processes may be lacking precision (Hagger et al., 2015) and some found comprehension and recall problems in participants’ responses to self-report habit measures (Gardner and Tang, 2014). Others, however, argue that individuals are able to reflect on automatically occurring behaviors and can interfere habit from its salient consequences, the habitual behavior that they show, although they were not thinking about it (Verplanken and Orbell, 2003; Sniehotta and Presseau, 2012). Alternative measures of automaticity need to be developed in future research with a special focus on their feasibility in long-term studies. Another limitation concerning the methods are the single-item scales used in this study to measure automaticity, affective valence, and arousal. Given that valence and arousal turned out to be strongly correlated, it must be critically noted that the scales were not appropriate for differentiating between the two dimensions that are actually considered orthogonal in the circumplex model (Russell, 1980). One explanation for this could be that we failed to explain the not very intuitive concept of arousal to the participants and, in particular, to describe its difference to valence (Ekkekakis and Petruzzello, 2002). Ekkekakis and Petruzzello (2002) note that exercise is able to change perceived activation and that these changes can lead to either positive or negative valence making it necessary to distinguish between the two dimensions of affect. However, since we were not interested in a differentiated pattern of affect as a dependent variable, but in this study focused on affect as a determinant of habit formation, the lack of specification appears to be negligible. Weighing the pros and cons of single-item measures, Ekkekakis and Petruzzello (2002) further mention that they pose a risk of random measurement error. However, important to our study was the assumption that given their compactness they do not induce reactivity to weekly testing. Regarding the measure for past exercise behavior used in this study, it can be critically mentioned that the definition of exercise given in the questionnaire was rather wide compared to the definition by Caspersen et al. (1985), which contains, different from our definition, the planned, structured, and repetitive nature of exercise.

### Implications

According to Gardner and Lally’s (2018) model of habit formation, individuals first need to form an intention when deciding to act; second, they need to initiate the action which requires mobilization of self-regulatory resources; third, they need to repeat the behavior for the strengthening of cue–response associations. In the present study, we focused on behavior repetition and affect as determinants of habit formation. Thus, the future research and practical implications that can be derived from this study and the literature that emphasizes the role of behavior repetition can be divided into two areas: Exercise promotion interventions and practitioners should design and implement interventions that result in (a) behavior repetition, and (b) a positive affective response to exercise. We suppose that the latter leads to behavior repetition. However, other important aspects of behavior maintenance include skills required to translate intentions into action, such as inclusion of self-monitoring in combination with other self-regulatory techniques, e.g., specific goal setting (Michie et al., 2009). In order to attain a goal, implementation intentions have been proven to have had a positive effect (Gollwitzer and Sheeran, 2006). Future studies should explore how to best design an exercise program that elicits regular positive affective responses in the participating individuals, as this is still one of the major challenges in this field. One possibility is to focus on the role of teachers or coaches for the development of positive affect of exercise class participants. The manipulation or education of teachers’ feedback (Leisterer and Jekauc, 2019), their leadership style (Raedeke et al., 2007), and their social-emotional skills (Strauch et al., 2018) are promising approaches. One study found four facilitators of positive emotional experiences of sport and exercise participants: perceived competence, perceived social interaction, novelty experience, and perceived physical exertion (Wienke and Jekauc, 2016). Furthermore, in one study, enjoyment after a theory-based “novel” physical education lesson that included evidence-based modifications, such as music, was higher than after a “traditional” physical education lesson, despite no significant differences in amount and intensity of PA components (Vazou et al., 2019). Future studies should investigate the relationship between affective states, behavior frequency, and habit formation by other measurements than self-reports and over a longer period of time, to explore the role of habits in long-term behavior maintenance. Since there is no such thing as a global physical activity habit (Gardner et al., 2020), this study focused on automaticity as an indicator of instigation habits. However, deeper understanding on the different habitual behavior sequences and their interplay with intention or other cognitive and automatic constructs is needed to progress further to a theory of habit that is still missing in the field.

## Conclusion

In conclusion, the present work discusses the importance of affective valence and behavior repetition in the formation of instigation habits in exercise contexts. Thus, interventions designed to encourage long-term behavior maintenance via habit formation processes, which are required for achieving sustainable health benefits, should try to elicit positive affective responses.

## Data Availability Statement

The raw data supporting the conclusions of this article will be made available by the authors, without undue reservation, to any qualified researcher.

## Ethics Statement

The studies involving human participants were reviewed and approved by the Ethik-Kommission der Universität Bielefeld (EUB), Ethics Committee of the Bielefeld University, Bielefeld University, Bielefeld, Germany. The patients/participants provided their written informed consent to participate in this study.

## Author Contributions

SW wrote the manuscript. SW, EF, and DJ conceptualized the study. EF and DJ organized the data collection. EF conducted the statistical calculations. All authors helped to edit the manuscript and approved the final version of the submitted manuscript.

## Conflict of Interest

The authors declare that the research was conducted in the absence of any commercial or financial relationships that could be construed as a potential conflict of interest.
